# Performance of the cuff leak test in adults in predicting post-extubation airway complications: a systematic review and meta-analysis

**DOI:** 10.1186/s13054-020-03358-8

**Published:** 2020-11-07

**Authors:** Akira Kuriyama, Jeffrey L. Jackson, Jun Kamei

**Affiliations:** 1 Emergency and Critical Care Center, Kurashiki Central Hospital, 1-1-1 Miwa, Kurashiki, Okayama 710-8602 Japan; 2 Division of General Internal Medicine, Department of Medicine, Medical College of Wisconsin, Milwaukee, USA

**Keywords:** Airway extubation, Airway obstruction, Cuff leak test, Guidelines, Sensitivity and specificity, Meta-analysis

## Abstract

**Background:**

Clinical practice guidelines recommend performing a cuff leak test in mechanically ventilated adults who meet extubation criteria to screen those at high risk for post-extubation stridor. Previous systematic reviews demonstrated excellent specificity of the cuff leak test but disagreed with respect to sensitivity. We conducted a systematic review and meta-analysis to assess the diagnostic accuracy of the cuff leak test for predicting post-extubation airway complications in intubated adult patients in critical care settings.

**Methods:**

We searched Medline, EMBASE, Scopus, ISI Web of Science, the Cochrane Library for eligible studies from inception to March 16, 2020, without language restrictions. We included studies that examined the diagnostic accuracy of cuff leak test if post-extubation airway obstruction after extubation or reintubation was explicitly reported as the reference standard. Two authors in duplicate and independently assessed the risk of bias using the Quality Assessment for Diagnostic Accuracy Studies-2 tool. We pooled sensitivities and specificities using generalized linear mixed model approach to bivariate random-effects meta-analysis. Our primary outcomes were post-extubation airway obstruction and reintubation.

**Results:**

We included 28 studies involving 4493 extubations. Three studies were at low risk for all QUADAS-2 risk of bias domains. The pooled sensitivity and specificity of cuff leak test for post-extubation airway obstruction were 0.62 (95% CI 0.49–0.73; *I*^2^ = 81.6%) and 0.87 (95% CI 0.82–0.90; *I*^2^ = 97.8%), respectively. The pooled sensitivity and specificity of the cuff leak test for reintubation were 0.66 (95% CI 0.46–0.81; *I*^2^ = 48.9%) and 0.88 (95% CI 0.83–0.92; *I*^2^ = 87.4%), respectively. Subgroup analyses suggested that the type of cuff leak test and length of intubation might be the cause of statistical heterogeneity of sensitivity and specificity, respectively, for post-extubation airway obstruction.

**Conclusions:**

The cuff leak test has excellent specificity but moderate sensitivity for post-extubation airway obstruction. The high specificity suggests that clinicians should consider intervening in patients with a positive test, but the low sensitivity suggests that patients still need to be closely monitored post-extubation.

## Background

Laryngeal edema and airway obstruction following extubation is a major cause of extubation failure [1]. Post-extubation stridor, its clinical sign, has a reported incidence of 2–26% and frequently results in reintubation [1]. Reintubation is associated with an increase in morbidity, duration of mechanical ventilation, and ICU stay [2–8]. In select cases, systemic corticosteroids before extubation can be used to prevent post-extubation airway complications [9]. Therefore, it is important to estimate the risk of laryngeal edema before extubation.

Since the endotracheal tube precludes direct visualization of the upper airway, the cuff leak test was proposed to predict the presence of laryngeal edema and post-extubation airway obstruction [10, 11]. Theoretically, when there is no laryngeal edema, there is an air leak around the tube after deflating the balloon cuff of the endotracheal tube [12, 13]. In contrast, a failed cuff leak test suggests little or no air leak around the tube, suggesting potential airway obstruction from laryngeal edema [12, 13]. The clinical practice guideline published by the American Thoracic Society and American College of Chest Physicians in 2017 recommends performing a cuff leak test in mechanically ventilated adults who meet extubation criteria to screen those at high risk for post-extubation stridor [14]. This guideline referenced two systematic reviews on the diagnostic accuracy of cuff leak test [15, 16], published in 2009 and 2011. Both reviews demonstrated excellent specificity of the cuff leak test but disagreed with respect to sensitivity. In addition, there have been several studies of the diagnostic accuracy of cuff leak test published after these reviews were completed.

Consequently, we conducted a systematic review and meta-analysis to assess the diagnostic accuracy of the cuff leak test for predicting post-extubation airway obstruction and subsequent reintubation.

## Methods

The conduct and reporting of this systematic review followed the PRISMA-DTA Statement [17]. Our review protocol was registered at PROSPERO (CRD42018084357).

We searched Medline, EMBASE, Scopus, ISI Web of Science, and the Cochrane Library for eligible studies from inception to March 16, 2020, without language restrictions [18]. Our search strategy was developed with the help of a medical librarian (Additional file 3: Table S1). We hand-searched the references of included articles for potentially relevant studies.

We examined the diagnostic accuracy of cuff leak test in intubated adult patients awaiting extubation in critical care settings. The index test was cuff leak test regardless of the type of cuff leak test (quantitative or qualitative) and threshold used. The reference standards included post-extubation airway obstruction determined by the original authors and subsequent reintubation. We included observational studies (cross-sectional and cohort studies) that examined the diagnostic accuracy of cuff leak test in critical care settings if: (1) the data were extractable into a 2 × 2 table from the reported data, (2) post-extubation airway obstruction after extubation or reintubation was explicitly reported as the reference standard. We considered both published studies and conference proceedings; however, we included the abstracts from conference proceedings only when they provided data in enough detail to be extractable. We considered interventional studies in critical care settings that examined the efficacy of systemic corticosteroids to prevent post-extubation airway complications; however, we excluded patients to whom systemic corticosteroids were administered after they were judged at high risk of post-extubation airway complications. Two authors (AK and JK) independently screened titles and abstracts obtained from the search and selected potentially relevant articles. Disagreement was resolved through discussion.

The first author (AK) and one of the other authors (JLJ and JK) in duplicate and independently extracted the following data from each study: (1) patient demographics (age, sex); (2) study characteristics (country, study population; duration of mechanical ventilation; mode of ventilation; observation period after extubation); (3) the type of cuff leak test (quantitative or qualitative); (4) numbers of true-positive, false-positive, true-negative, and false-negative; and (5) the reference standards used. Quantitative cuff leak test measures the air leak volume with a cuff deflated and judges the post-extubation airway obstruction based on its absolute volume or proportion in comparison with the expiratory tidal volume against a certain threshold [19]. Qualitative cuff leak test examines the presence or absence of audible expired air around an endotracheal tube, which indicates the pass or failure of the test [19]. In this study, a lack of a cuff leak, having a risk of post-extubation complications, was considered a positive test, while having a leak, suggesting low risk of post-extubation complications was a negative test [19].

Two authors (AK and JLJ) independently assessed the risk of bias using the Quality Assessment for Diagnostic Accuracy Studies-2 (QUADAS-2) tool [20]. Inconsistency was resolved through consensus.

### Statistical analysis

We had two primary outcomes: (1) post-extubation airway obstruction and (2) reintubation due to post-extubation airway obstruction. The reference standards for post-extubation airway obstruction included stridor (audible high-pitched inspiratory wheeze) [21], or laryngeal edema defined by the study authors (including confirmation with bronchoscopy [22] or laryngoscope [23]).

We pooled the data using a generalized linear mixed model approach to bivariate random-effects meta-analysis to calculate summary estimates of sensitivity, specificity, and likelihood ratios as well as the associated 95% confidence intervals (CIs) [24]. We pooled prevalence using a random-effects model, with exact binomial estimates of standard deviation and the Freeman–Tukey transformation for zero cells [25]. To examine the sources of heterogeneity, we examined the receiver operating characteristic (ROC) curves, assessed the correlation between sensitivity and specificity, and analyzed whether sensitivity and specificity of cuff leak changed with the type of cuff leak test (qualitative or quantitative), the proportion of women, inclusion or exclusion of reintubated patients in a study, and the length of intubation, using subgroup or meta-regression analysis [14, 19]. We also calculated the sensitivity and specificity of cuff leak test using a cutoff of 110 mL, a value that is frequently used in clinical practice [21]. We tested for publication bias using Deeks’ method [26]. We created a Fagan's nomogram, which determines the posttest probability according to the pretest probability and the calculated positive and negative likelihood ratios [27]. We followed standard diagnostic meta-analytic approaches in focusing on the sensitivity, specificity, and likelihood ratios instead of positive and negative predictive values because predictive values are dependent on the population prevalence of post-extubation complications, which can vary considerably. The threshold of statistical significance was set at *P* < 0.05. All analyses were performed with Stata SE, version 15.1 (Stata Corp; College Station, TX).

## Results

**Fig. 1:**
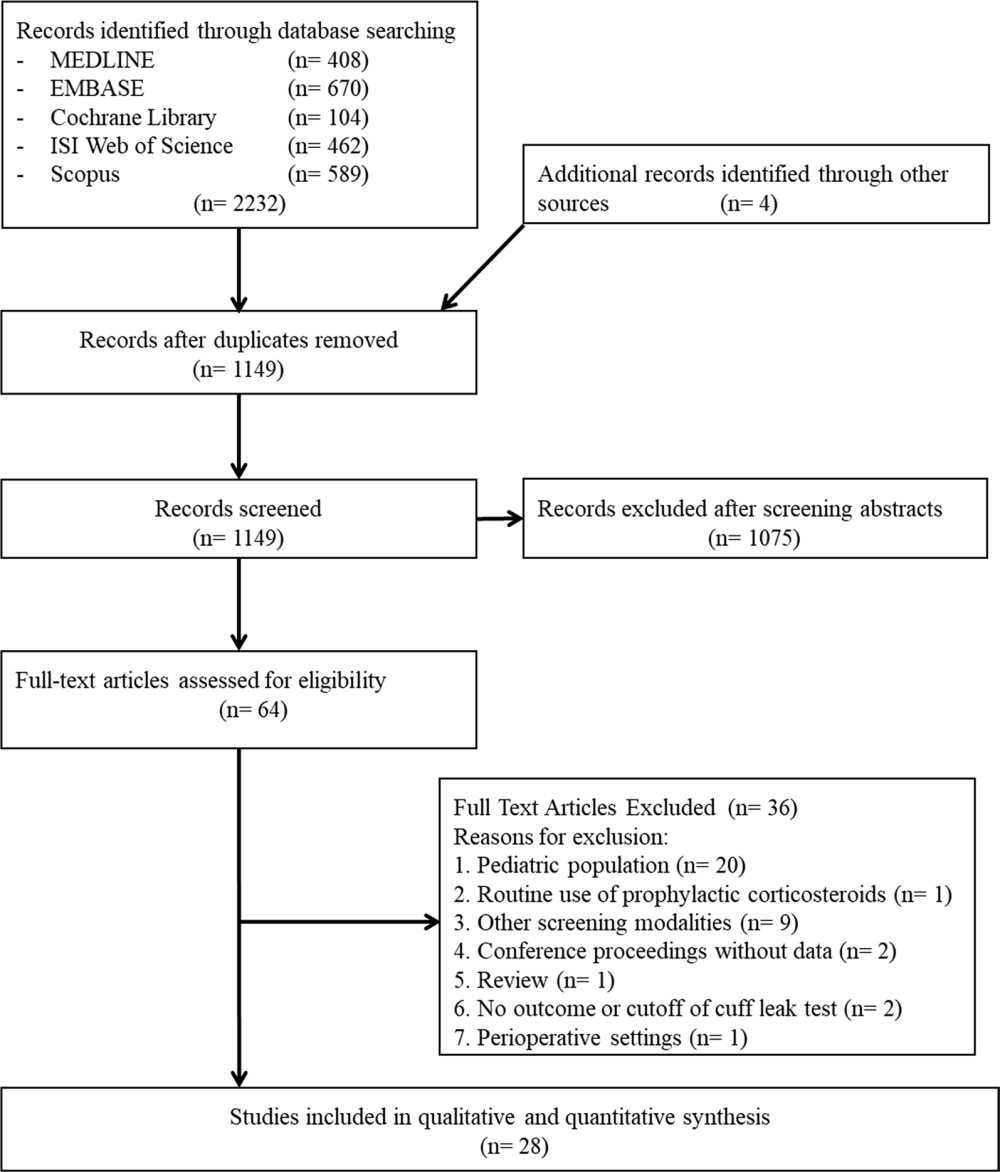
Flowchart of study selection

Our literature search produced 2236 studies. After application of inclusion and exclusion criteria, 28 studies involving 4493 extubations were included in the analysis (Fig. 1) [12, 13, 21–23, 28–50]. Among the 28 included studies, 27 were published in English and one in Korean [35].

Twenty-seven studies (96%) were prospective [12, 13, 21–23, 28–37, 39–50]. Nine studies and ten were conducted in medical [21, 22, 31, 32, 35–37, 43, 49] and mixed intensive care units [12, 13, 23, 28, 33, 34, 40, 41, 44, 45], respectively (Table 1). The included studies were published between 1992 and 2019. All but one were published studies and the remaining one an abstract in a conference proceeding. Eight studies were conducted in the USA, four each in France and Taiwan, three in Thailand, two in Egypt, one each in Australia, Belgium, India, Iran, Italy, South Korea, and Turkey. The median sample size was 101, ranging 34–543 (interquartile range 51–236). The reported mean/median duration of mechanical ventilation ranged from 2 to 28.1 days.

**Table 1:** Characteristics of participants in the included studies

Author/year	Country	Study population	No. of extubation (%, female)	No. of extubations	Age	Duration of mechanical ventilation (days)	Inclusion of unplanned extubation	Exclusion of reintubated patients
Fisher/1992 [23]	Australia	Mixed ICU	62 (NR)	62	NR	NR	Unclear	Yes
Marik/1996 [28]	USA	Mixed ICUs	100 (39)	100	57	3.8	Unclear	Yes
Miller/1996 [21]	USA	Medical ICU	100 (58)	100	63	5.8	Yes	Yes
Engoren/1999 [29]	USA	Cardiovascular ICU	524 (33.0)	531	65	12.9	Unclear	No
Sandhu/2000 [30]	USA	Trauma ICU	110 (27.2)	110	NR	3.0	No	Yes
De Bast/2002 [12]	Belgium	Mixed ICU	76 (NR)	76	67^a^	2^a^	Unclear	Yes
Jaber/2003 [13]	France	Mixed ICU	112 (30.4)	112	59.2	6.1	No	Yes
Maury/2004 [31]	France	Medical ICU	99 (47.4)	115	60	3.5	No	No
Erginel/2005 [32]	Turkey	Respiratory (medical) ICU	56 (16.4)	67	63.6	5.6	Unclear	No
Kriner/2005 [33]	USA	Mixed ICUs	462 (46.8)	462	61	5	No	Yes
Cheng/2006 [34]	Taiwan	Mixed ICU	236 (NR)	236	NR	NR	No	Yes
Chung/2006 [22]	Taiwan	Medical ICU	95 (33.7)	95	71.3	28.1	Yes	Yes
Lim/2006 [35]	South Korean	Medical ICU	34 (32.4)	34	60.4	4.7	Unclear	Yes
Lee/2007 [36]	Taiwan	Medical ICU	325 (NR)	325	NR	NR	No	Yes
Wang/2007 [37]	Taiwan	Medical ICU	110 (52.7)	110	71	13	No	Yes
Shin/2008 [38]	USA	Burn/Trauma ICU	49 (32.7)	49	36.8	3.3	Unclear	Yes
Sukhupanyarek/2008 [39]	Thailand	Not specified	543 (40.3)	543	60.3	4.0	Yes	Yes
Antonaglia/2010 [40]	Italy	Mixed ICU	42 (52.4)	42	63.3	6^a^	Yes	Yes
Gros/2012 [41]	France	Mixed ICU	104 (40.4)	104	10^a^	Stridor: 5^a^ Non-stridor: 4^a^	No	Yes
Keeratichananont/2012 [42]	Thailand	Not specified	115 (47.0)	115	57.8	5.8	No	Yes
Radhi/2012 [43]	USA	Medical ICU	51 (39.2)	51	56^a^	3^a^	Unclear	Yes
Sutherasan/2013 [44]	Thailand	Mixed ICU	101 (38.6)	101	67.8	6.5	Unclear	Yes
Mikaeili/2014 [49]	Iran	Neurology/Medical ICUs	41 (39.0)	41	57.2	NR	Unclear	Yes
Patel/2015 [45]	USA	Mixed ICUs	51 (41.2)	51	NR	3.8	Unclear	Yes
El-Baradey/2016 [46]	Egypt	Not specified	432 (26.9)	432	45.0	10.1	Unclear	Yes
Sahbal/2016 [47]	Egypt	Not specified	50 (38)	50	NR	NR	Unclear	Yes
Schnell/2017 [48]	France	Not specified	362 (40.6)	362	60^a^	5^a^	No	Yes
Samanta/2019 [50]	India	Not specified	51 (44.2)	51	39.6	7.5	Unclear	Unclear

*ICU* intensive care unit, *NR* not reported, *USA* United States of America

^a^Variables were shown in median

Five studies used a qualitative cuff leak test (auscultation of airflow) [23, 28, 31, 39, 45], and 21 used a quantitative measurement of the cuff leak [12, 13, 21, 22, 29, 30, 32–37, 40–44, 46, 47, 49, 50] (Table 2). One study reported the results from both qualitative and quantitative cuff leak tests examined in a single cohort [48]. The remaining study reported on the data of patients who underwent either qualitative or quantitative cuff leak test [38]. The most frequent cutoff values for quantitative cuff leak tests ranged from 50 to 283 mL (median, 110 mL) in volume and from 10 to 57% in proportion. Twenty studies used assist control ventilation [12, 13, 21, 22, 29, 30, 32–38, 41–44, 46, 47, 49] , and four used spontaneous breathing including pressure support ventilation [23, 31, 40, 45]. Another study examined either of these two ventilation modes [28]. One study applied the ambu bag, while the cuff leak was tested [39]. One study performed a qualitative cuff leak test during spontaneous breathing via T-tube and during cough while still intubated [48]. The remaining one did not report the mode of ventilation used [50]. Twenty-four studies used stridor [12, 13, 21, 28–39, 41–43, 45–50] and three used direct visualization of the airway following extubation as reference standards [22, 23, 40] for airway obstruction. One study used either of these reference standards [44].

**Table 2:** Summary of cuff leak test and reference standards in the included studies

Author/year	Type of cuff leak test	Mode of mechanical ventilation	Tidal volume	Cutoff	Diagnosis/sign of upper airway obstruction	Observation period (hours)	Incidence of upper airway obstruction, % (events/total)
Fisher/1992 [23]	Qualitative	Spontaneous ventilation	NR	Presence or absence of peritubular leak	Direct laryngoscopy	NR	11.3 (7/62)
Marik/1996 [28]	Qualitative	Spontaneous or positive pressure ventilation	NR	Presence or absence of peritubular leak	Stridor	24	2 (2/100)
Miller/1996 [21]	Quantitative	Assist control	NR	110 mL	Stridor	NR	6 (6/100)
Engoren/1999 [29]	Quantitative	Assist control	10- 12 mL/kg	110 mL	Stridor	NR	0.6 (3/531)
Sandhu/2000 [30]	Quantitative	Assist control	NR	10%	Stridor	24	11.8 (13/110)
De Bast/2002 [12]	Quantitative	Assist control	NR	15.5%	Stridor	24	13.2 (10/76)
Jaber/2003 [13]	Quantitative	Assist control	10- 12 mL/kg	130 mL/12%	Stridor	48	11.6 (13/112)
Maury/2004 [31]	Qualitative	Spontaneous breathing via T-tube	NR	Presence or absence of respiratory flow	Stridor	24	3.5 (4/115)
Erginel/2005 [32]	Quantitative	Assist control	7 mL/kg	283 mL/57%	Stridor	NR	10.4 (7/67)
Kriner/2005 [33]	Quantitative	Assist control	NR	110 mL/15.5%	Stridor	24	4.3 (20/462)
Cheng/2006 [34]	Quantitative	Assist control	8 mL/kg	24%	Stridor	48	7.6 (18/236)
Chung/2006 [22]	Quantitative	Assist control	10 mL/kg	140 mL	Laryngeal edema based on video bronchoscopy	NR	36.8 (35/95)
Lim/2006 [35]	Quantitative	Assist control	8- 10 mL/kg	50 mL/14.7%	Stridor	24	8.8 (3/34)
Lee/2007 [36]	Quantitative	Assist control	10 mL/kg	110 mL	Stridor	48	7.7 (25/325)
Wang/2007 [37]	Quantitative	Assist control	10 mL/kg	88 mL/18%	Stridor	NR	18.2 (20/110)
Shin/2008 [38]	Quantitative or qualitative	Assist control	10 mL/kg	10% or audible air expired	Stridor	NR	2.0 (1/49)
Sukhupanyarek/2008 [39]	Qualitative	Used an ambu bag	NR	Presence or absence of audible leak	Stridor	24	4.8 (26/543)
Antonaglia/2010 [40]	Quantitative	Spontaneous ventilation	NR	70 mL (21%)	Laryngeal lesions based on a rigid laryngoscope	NR	4.8 (2/42)
Gros/2012 [41]	Quantitative	Assist control	10 mL/kg	130 mL	Stridor	48	6.7 (7/104)
Keeratichananont/2012 [42]	Quantitative	Assist control	500 mL	114 mL	Stridor	72	16.5 (19/115)
Radhi/2012 [43]	Quantitative	Assist control	6-8 mL/kg	15%	Stridor	1	7.8 (4/51)
Sutherasan/2013 [44]	Quantitative	Assist control	10 mL/kg	110 mL	Stridor or laryngoscopy finding (erythematous swell of vocal cords)	NR	16.8 (17/101)
Mikaeli/2014 [49]	Quantitative	Assist control	NR	110, 130, 249 mL	Stridor	24	9.8 (4/41)
Patel/2015 [45]	Qualitative	Spontaneous breathing	NR	Presence or absence of audible leak	Stridor	24	3.9 (2/51)
El-Baradey/2016 [46]	Quantitative	Assist control	8 mL/kg	200 mL	Stridor	24	10.5 (45/432)
Sahbal/2016 [47]	Quantitative	Assist control	NR	132.5 mL	Stridor	NR	8 (4/50)
Schnell/2017 [48]	Quantitative/Qualitative	Assist control/spontaneous breathing via T-tube/cough during spontaneous breathing	8 mL/kg	Presence or absence of audible leak/110 mL	Stridor	48	9.4 (34/362)
Samanta/2019 [50]	Quantitative	NR	NR	110 mL	Stridor	NR	21.2 (11/52)

*NR* not reported

Three of the 28 studies were at low risk of bias for all QUADAS-2 risk of bias domains (Table 3). Seventeen studies (60.7%) were deemed at low risk of bias for the domain of patient selection. Eight out of 22 studies that assessed quantitative cuff leak prespecified the cutoff of cuff leak; fourteen studies (50%) were deemed to have adequately assessed the index test. A reference standard was adequately assessed in 18 studies (64.3%). Study participants were adequately followed up in 19 studies (67.9%).

**Table 3:** Evaluation of the included studies with quality assessment of diagnostic accuracy studies-2

Author/year	Risk of bias				Applicability concerns		
	Patient selection	Index test	Reference standard	Flow and timing	Patient selection	Index test	Reference standard
Fisher/1992 [23]	Unclear	Low	High	High	Low	Unclear	High
Marik/1996 [28]	Low	Low	Low	Low	Low	Low	Low
Miller/1996 [21]	Low	High	Low	Low	Low	Low	Low
Engoren/1999 [29]	Unclear	Low	Low	Unclear	Low	Low	Low
Sandhu/2000 [30]	Low	Unclear	Low	Low	Low	Low	Low
De Bast/2002 [12]	Low	High	Low	Low	Low	Low	Low
Jaber/2003 [13]	Unclear	High	Low	Low	Low	Low	Low
Maury/2004 [31]	Low	Low	Low	Low	Low	Low	Low
Erginel/2005 [32]	Low	Low	Low	Unclear	Low	Low	Low
Kriner/2005 [33]	Low	Low	Unclear	Low	Low	Low	Low
Cheng/2006 [34]	Low	Low	Unclear	Unclear	Low	Low	Low
Chung/2006 [22]	High	High	Low	Low	Low	Low	Low
Lim/2006 [35]	Low	High	Unclear	Low	Low	Low	Low
Lee/2007 [36]	Low	Low	Unclear	Unclear	Low	Low	Low
Wang/2007 [37]	Low	High	Low	Low	Low	Low	Low
Shin/2008 [38]	Unclear	Unclear	Unclear	Unclear	Low	Unclear	Unclear
Sukhupanyarek/2008 [39]	Unclear	Low	Low	Low	Low	Low	Low
Antonaglia/2010 [40]	Low	High	Low	Low	Low	Low	Low
Gros/2012 [41]	Low	High	Unclear	Low	Low	Low	Low
Keeratichananont/2012 [42]	Unclear	Low	Low	Unclear	Low	Low	Unclear
Radhi/2012 [43]	Unclear	Low	Low	Low	Low	Low	Low
Sutherasan/2013 [44]	Unclear	Unclear	Low	Low	Low	Low	Low
Mikaeli/2014 [49]	Low	Low	Low	Low	Low	Low	Low
Patel/2015 [45]	Low	Low	Unclear	Low	Low	Low	Low
El-Baradey/2016 [46]	Low	High	Low	Low	Low	Low	Low
Sahbal/2016 [47]	Low	High	Unclear	Unclear	Low	Low	Low
Schnell/2017 [48]	Unclear	Low	Low	Low	Low	Low	Low
Samanta/2020 [50]	Unclear	Unclear	Unclear	Unclear	Unclear	Unclear	Unclear

### Post-extubation airway obstruction

**Fig. 2:**
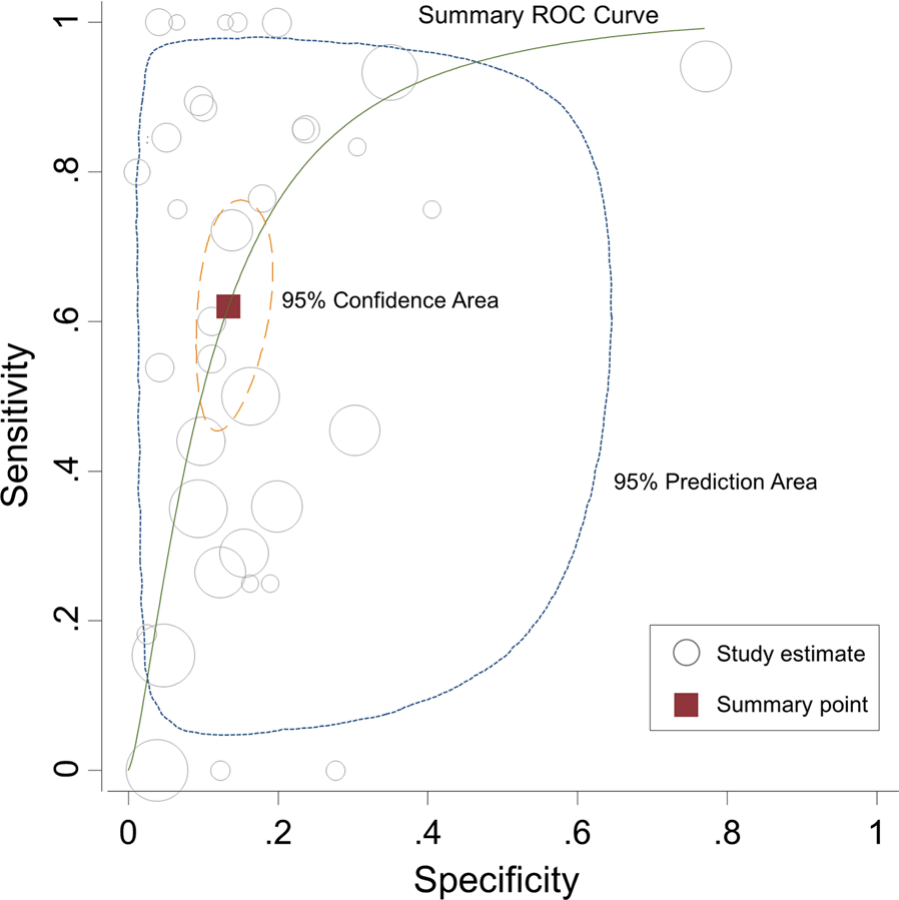
Summary receiver operating characteristics (SROC) curve of the cuff leak test for predicting post-extubation upper airway obstruction. The circle size represents the number of extubation in each study. The pooled sensitivity is 0.62 (95% CI 0.49–0.73) and the pooled specificity is 0.87 (95% CI 0.82–0.90), with the area under the SROC curve of 0.85 (95% CI 0.82–0.88). The prediction region illustrates the extent of statistical heterogeneity by depicting a region within there is 95% confidence that the true sensitivity and specificity of a future study should lie

The prevalence of post-extubation airway obstruction ranged from 4 to 37% (pooled estimate 9%; 95% CI 7–11; *I*^2^ = 86.3%). The pooled sensitivity and specificity of cuff leak test for post-extubation airway obstruction were 0.62 (95% CI 0.49–0.73; *I*^2^ = 81.6%) and 0.87 (95% CI 0.82–0.90; *I*^2^ = 97.8%), respectively. The forest plots are shown in Additional file 1: Figure S1. The pooled positive and negative likelihood ratios were 4.63 (95% CI 3.44–6.22) and 0.44 (95% CI 0.32–0.60), respectively. The area under the summary receiver operating characteristic (SROC) curve was 0.85 (95% CI 0.82–0.88) (Fig. 2), and the pooled diagnostic odds ratio (DOR) was 10.54 (95% CI 6.26–17.76) (Additional file 4: Table S2). There was no evidence of publication bias (*p* = 0.189).

Subgroup analysis suggested that the specificity was similar between the qualitative and quantitative cuff leak tests (qualitative 0.89 [95% CI 0.82–0.96] vs. quantitative 0.86 [95% CI 0.81–0.91], *p* < 0.01). While the sensitivity difference was clinically important, it was statistically not significant (quantitative 0.67 [95% CI 0.56–0.78] vs. qualitative 0.35 [95% CI 0.12–0.57], *p* = 0.07). The specificity of the cuff leak test was slightly but significantly (*p* = 0.02) worse in patients intubated more than 6 days (0.85 [95% CI 0.76–0.95]) than those intubated ≤ 6 days (0.87 [95% CI 0.81–0.92]), with no significant difference in the sensitivity. There was no difference of sensitivity or specificity with the proportion of women or with the exclusion of reintubated patients. The sensitivity and specificity of cuff leak test based on a cut point of 110 mL were 0.44 (95% CI 0.31–0.59) and 0.91 (95% CI 0.82–0.95), respectively.

**Fig. 3:**
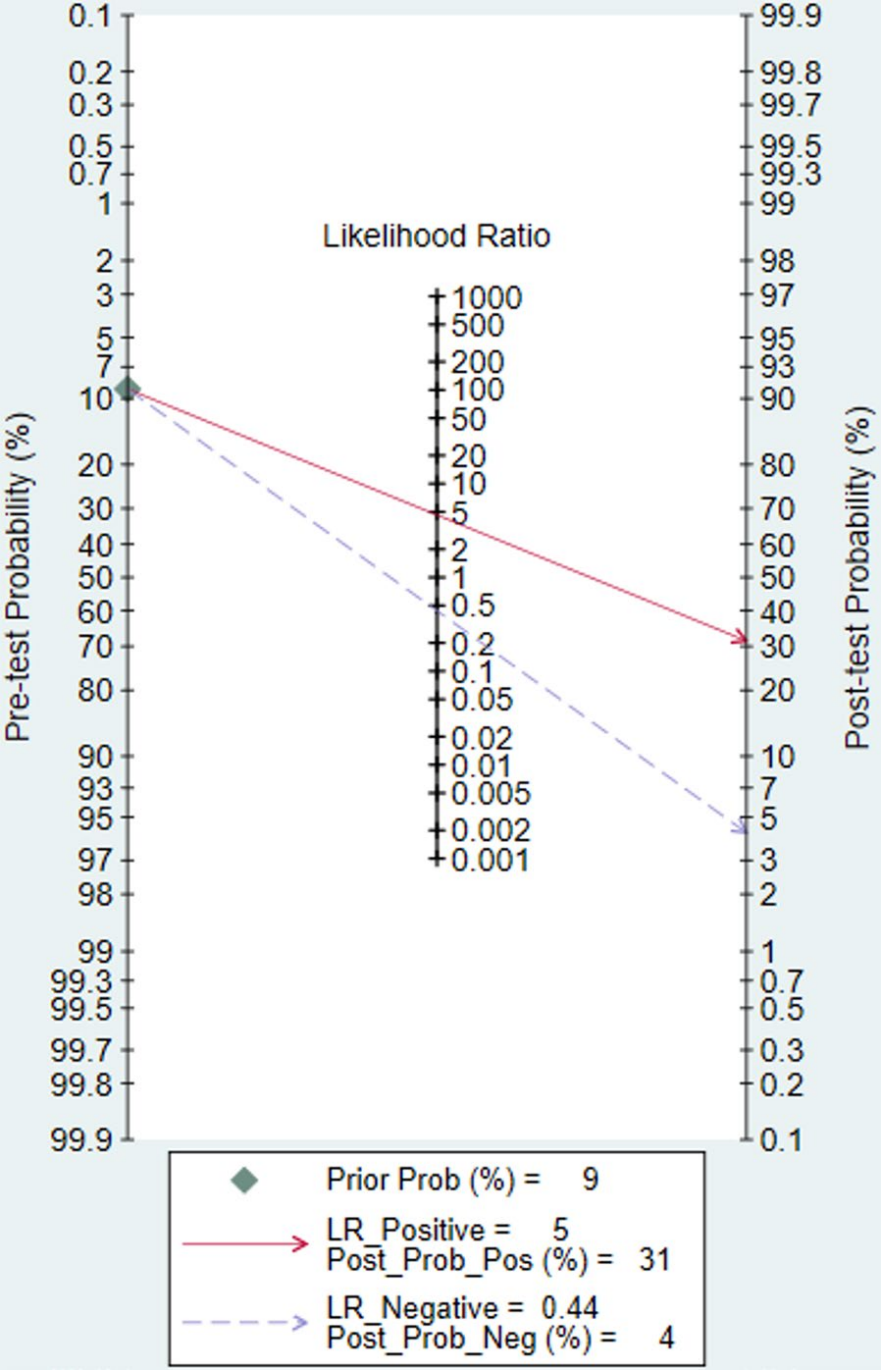
A Fagan’s nomogram for predicting post-extubation upper airway obstruction

A nomogram based on the pretest probability of 9% (the incidence of stridor in the studies included in our study) is provided (Fig. 3).

### Reintubation

**Fig. 4:**
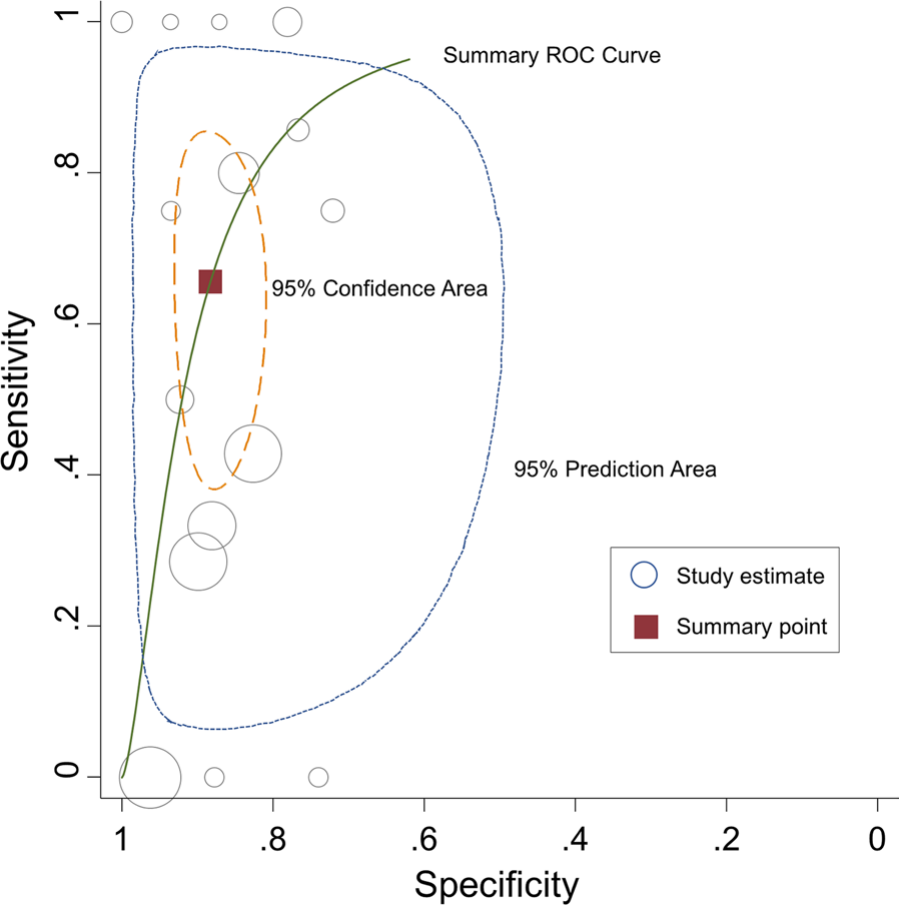
Summary receiver operating characteristics (SROC) curve of the cuff leak test for predicting reintubation. The circle size represents the number of extubation in each study. The pooled sensitivity is 0.66 (95% CI 0.46–0.81) and the pooled specificity is 0.88 (95% CI 0.83–0.92), with the area under the SROC curve of 0.88 (95% CI 0.83–0.90). The prediction region illustrates the extent of statistical heterogeneity by depicting a region within there is 95% confidence that the true sensitivity and specificity of a future study should lie

The prevalence of reintubation varied from 0 to 11% (pooled estimate: 3%; 95% CI 1–5; *I*^2^ = 79%). The pooled sensitivity and specificity of the cuff leak test for reintubation were 0.66 (95% CI 0.46–0.81; *I*^2^ = 48.9%) and 0.88 (95% CI 0.83–0.92; *I*^2^ = 87.4%), respectively. The forest plots of the sensitivity and specificity of the cuff leak test for predicting reintubation are shown in Additional file 2: Figure S2. The pooled positive and negative likelihood ratios were 5.59 (95% CI 3.48–8.98) and 0.39 (95% CI 0.23–0.66). The area under the SROC curve was 0.88 (95% CI 0.83–0.90) (Fig. 4) and the pooled DOR was 14.34 (95% CI 5.65–36.42) (Additional file 4: Table S2). There was no evidence of publication bias (*p* = 0.52).

## Discussion

Our study found that the cuff leak test has excellent specificity but moderate sensitivity for post-extubation airway obstruction. The cuff leak test thus works better to rule in than to rule out potential post-extubation airway obstruction. However, the false-negative rate of 38% suggests that the cuff leak test may fail to identify some patients with post-extubation airway obstruction.

Our study found that the specificity of the cuff leak test for post-extubation airway obstruction was excellent, which is consistent with two previous systematic reviews [15, 16]. In contrast, Ochoa et al. and Zhou et al. concluded that the sensitivity of cuff leak testing for post-extubation airway obstruction was 56% and 80%, respectively [15, 16]: Our pooled sensitivity was 62%, which fell between those two findings. We included nearly double the number of studies that their reviews did. Furthermore, the additional studies we included were higher quality, potentially making our findings more reliable.

Our analysis found that the qualitative cuff leak test had low sensitivity (35%) in predicting post-extubation airway obstruction. This has been consistently found in recent studies. The most likely explanation is the subjective nature of this test. In addition, since Schnell et al. provided data from three different methods of qualitative cuff leak testing [48], the sensitivity of which were all around 30%; this study may have been overweighed, although repeat analysis limiting Schnell’s study to a single data contribution did not change the sensitivity of the qualitative test. In contrast, the specificity of both qualitative and quantitative cuff leak tests was high, nearly 90%, while there was a statistically significant difference between two methods, clinically both performed equally well. A cutoff of 110 mL also had a low sensitivity (44%) and high specificity for predicting post-extubation airway obstruction. We thus conclude that the cuff leak test has high specificity and can be used to select patients to consider treating with systemic corticosteroids, but its low sensitivity suggests that the traditional practice of closely observing all patients in the immediate post-extubation period should be continued. Consistent with these findings, the nomogram suggested that while a negative cuff leak test represents low possibility of post-extubation airway obstruction, a positive test still provides a relatively low posttest probability.

The guideline by ATS/ACCP provided a conditional recommendation regarding cuff leak test [14], because failing the cuff leak test might lead a delay in extubation and an increase in complications such as barotrauma and ventilator-associated pneumonia. The guideline weakly recommended that the cuff leak test be reserved for high-risk patients, who experienced a traumatic intubation, were intubated more than 6 days, have a large endotracheal tube, are female, or were reintubated after an unplanned extubation [14]. Our analysis found that the length of intubation had a small impact on the specificity of cuff leak test. Female sex and reintubation had no impact of the accuracy of cuff leak test. Since the original studies included in our review examined non-selected patients with respect to the risk of post-extubation airway obstruction and the sensitivity of cuff leak test is moderate, we support the idea of the ATS/ACCP guideline to reserve cuff leak test for high-risk patients.

Our study suggested that the cuff leak test has moderate sensitivity and excellent specificity for reintubation. Although the sensitivity in our study was similar to those of previous meta-analyses, the specificity in our study was slightly higher [15, 16]. The area under the SROC curve and DOR were also greater than previously reported [16]. Thus, a failed cuff leak test may serve as a good marker for those at risk of reintubation, if post-extubation airway obstruction is not treated adequately.

The limitation of cuff leak test has been repeatedly discussed. Cuff leak test can be susceptible to relationship of tube size to laryngeal diameter [41], respiratory system compliance and resistance, inspiratory flow, expiatory flow and time, and airway collapse [51], and clinicians should bear in mind that the ability of cuff leak test may vary according to the condition or type of patients [52]. Additionally, coughing during cuff deflation test hinders accurate measurement of the leak volume and lowers the reproducibility. A previous physiological study suggested that while patients were sedated and paralyzed, the cuff leak volume was reliably measurable [53]. An adequate amount of sedatives and opioids can suppress coughing during the airway suctioning before cuff leak test or cuff deflation during the test. Further, cuff leak testing is recommended several hours before extubation, which allows the arousal of patients from sedation by the time of extubation. Thus, we may be able to at least attempt to increase the reliability of cuff leak measurement.

Few tests are available to estimate the risk of post-extubation airway complications. A case series of three patients suggested that video laryngoscopy enabled visualization of laryngeal edema prior to extubation [54], but its clinical efficacy in estimating post-extubation airway complications is yet to be determined. Several studies have examined the role of laryngeal ultrasonography in adult patients. Laryngeal air column width difference is the difference between width of airway at the level of the vocal cord with cuff inflated and deflated. Its reported sensitivity and specificity varied across studies, ranging from 50 to 91% and 54 to 72%, respectively [44, 46, 49]. Laryngeal air column width ratio is the ratio of air column width before extubation over that after intubation. It has been examined in only one study [55] and needs further validations. Thus, no single available options can correctly estimate the risk of post-extubation airway complications. Clinicians should not overly rely on one single test in predicting the success or failure of extubation.

Our study had several strengths and limitations. Strengths included a comprehensive search in five databases without language restrictions. This allowed us to conduct relevant subgroup analyses with a larger number of studies. Further, inclusion of non-English studies facilitates the generalizability of our findings in various clinical settings [56].

Our study had some limitations. First, the definition of post-extubation airway obstruction differed across studies. Stridor was more frequently used as the reference standards than laryngeal edema (as assessed with endoscopy). Laryngeal edema may be more frequent than stridor, because stridor and respiratory distress occur when laryngeal edema narrows the airway by ≥ 50% [57]. However, laryngeal edema is not always screened for in extubated patients, and the presence of stridor is an accepted sign of respiratory distress that triggers a concern for airway obstruction. Thus, the finding of our study is generalizable to the clinical practice. Second, although we attempted to include in the analysis the incidence of stridor due to post-extubation airway events, some patients might have had a concurrent clinical state that necessitated high minute ventilation or tachypnea through an edematous airway, which manifested as ‘stridor.’ Therefore, we might not have been able to completely separate stridor due to post-extubation airway events from stridor due to other etiologies, such as respiratory insufficiency. This limitation also applies to reintubation. Third, whether to reintubate patients is subject to treating physicians’ discretion and the effect of treatment to abort post-extubation stridor. Therefore, the value of cuff leak test in predicting the need for reintubation in clinical practice may be limited along with the third limitation. However, prevention of post-extubation airway obstruction is more important than reintubation per se. Once the cuff leak test identifies patients at high risk of post-extubation airway obstruction, prophylactic systemic corticosteroids are indicated [9, 14, 58]. Fourth, 15 out of 23 studies that assessed the quantitative cuff leak test determined the cutoff with the knowledge of the results of the reference standards. It is known that data-driven optimization of the cutoff can lead to overestimation of test performance [59]. Thus, the pooled accuracy of quantitative cuff leak test in our study can be an overestimation; the optimal cutoff is still unknown. Fifth, the quantitative cutoff for a positive test varied between the studies. Since we had aggregate data from each included study, we failed to determine the optimal cutoff of cuff leak test. Finally, there was substantial statistical heterogeneity in the pooled sensitivity and specificity for both outcomes. The presence of statistical heterogeneity is a common issue intrinsic to the meta-analysis of diagnostic accuracy of a test, given the clinical and methodological diversity in original studies as well as the possible relationship between sensitivity and specificity, as exemplified in ROC curves in which more sensitive cut points have lower specificity (and vice versa). Our subgroup analyses suggested that the type of cuff leak test (quantitative versus qualitative) and length of intubation might have been the cause of statistical heterogeneity of sensitivity and specificity, respectively, for post-extubation airway obstruction.

## Conclusion

The cuff leak test has excellent specificity but moderate sensitivity for post-extubation airway obstruction. The cuff leak test is a useful tool in the decision-making about extubation, but the low sensitivity suggests that a negative test cannot completely exclude post-extubation airway obstruction and that patients still need to be closely monitored post-extubation. The higher specificity suggests that clinicians should consider intervening in patients with systemic corticosteroids in response to a positive test. Continued research to find better modalities to rule out post-extubation airway obstruction is needed.

## Supplementary information

**Additional file 1: Figure S1.** Forest plot of the sensitivity and specificity of the cuff leak test for predicting post-extubation upper airway obstruction.

**Additional file 2: Figure S2.** Forest plot of the sensitivity and specificity of the cuff leak test for predicting reintubation.

**Additional file 3: Table S1.** Search strategy.

**Additional file 4: Table S2.** The pooled diagnostic accuracy of cuff leak test for post-extubation airway obstruction and reintubation.

## Data Availability

All data generated or analyzed during this study are included in this published article and its supplementary information files.
