# Supplementary material for: Performance of the cuff leak test in adults in predicting post-extubation airway complications: a systematic review and meta-analysis
Source: Crit Care. 2020 Nov 7;24:640. doi: 10.1186/s13054-020-03358-8 (PMC7648377; doi:10.1186/s13054-020-03358-8)
Supplement: Supplementary file 3 — Additional file 3: Table S1. Search strategy. [file 13054_2020_3358_MOESM3_ESM.docx]

**Additional file 3: Table S1**. Search Strategy.

**Medline**

#1. ("air leak*" or air-leak).mp. or ((Intubation, Intratracheal/ or Intratracheal intubation.mp.) and leak*.mp. and (measur* or instrument* or test* or exam*).mp.)

#2. (stridor or extubat*).mp.

#3. #1 AND #2

#4. ((cuff adj3 leak*) or cuff-leak*).mp.

#5. #3 OR #4

**EMBASE**

#1. ('air leak*' OR 'air-leak' OR (('endotracheal intubation'/exp OR 'endotracheal intubation' OR 'intratracheal intubation'/exp OR 'intratracheal intubation') AND leak* AND (measur* OR instrument* OR test* OR exam*))

#2. stridor OR extubat*

#3. #1 AND #2

#4. ((cuff NEAR/3 leak*) OR 'cuff leak*')

#5. #3 OR #4

**Scopus**

TITLE-ABS-KEY ( ( ( ( intratracheal AND intubation AND leak* ) AND ( measur* OR instrument* OR test OR tests OR exam* ) ) OR ( "air leak*" OR air-leak ) ) AND ( stridor OR extubat* ) ) OR TITLE-ABS-KEY ( cuff W/3 leak* )

**Web of Science**

TS = ( ( ( ( intratracheal AND intubation AND leak* ) AND ( measur* OR instrument* OR test OR tests OR exam* ) ) OR ( "air leak*" OR air-leak ) ) AND ( stridor OR extubat* ) ) OR TS = ( cuff NEAR/3 leak* )

**The Cochrane Library**

((("air leak*") OR ("intratracheal intubation" AND leak*)) AND (extubat* OR stridor)) OR ("cuff leak*")
