# Supplementary material for: Performance of the cuff leak test in adults in predicting post-extubation airway complications: a systematic review and meta-analysis
Source: Crit Care. 2020 Nov 7;24:640. doi: 10.1186/s13054-020-03358-8 (PMC7648377; doi:10.1186/s13054-020-03358-8)
Supplement: Supplementary file 4 — Additional file 4: Table S2. The pooled diagnostic accuracy of cuff leak test for post-extubation airway obstruction and reintubation. [file 13054_2020_3358_MOESM4_ESM.docx]

**Additional file 4: Table S2**. The pooled diagnostic accuracy of cuff leak test for post-extubation airway obstruction and reintubation.

|  | Pooled diagnostic accuracy  (95% confidence interval) |
| --- | --- |
| Post-extubation airway obstruction | |
| Sensitivity | 0.62 (0.49 to 0.73) |
| Specificity | 0.87 (0.82 to 0.90) |
| Positive likelihood ratio | 4.63 (3.44 to 6.22) |
| Negative likelihood ratio | 0.44 (0.32 to 0.60) |
| DOR | 10.54 (6.26 to 17.76) |
| Reintubation | |
| Sensitivity | 0.66 (0.46 to 0.81) |
| Specificity | 0.88 (0.83 to 0.92) |
| Positive likelihood ratio | 5.59 (3.48 to 8.98) |
| Negative likelihood ratio | 0.39 (0.23 to 0.66) |
| DOR | 14.34 (5.65 to 36.42) |

Abbreviation; DOR, diagnostic odds ratio.
